# Assessing the feasibility of Phase 3 vaccine trials against Marburg Virus Disease: A modelling study

**DOI:** 10.1016/j.jvacx.2023.100321

**Published:** 2023-06-09

**Authors:** George Y. Qian, Thibaut Jombart, W. John Edmunds

**Affiliations:** a Department of Engineering Mathematics, University of Bristol, United Kingdom; b Centre for Mathematical Modelling of Infectious Diseases, London School of Hygiene & Tropical Medicine, London, United Kingdom; c MRC Centre for Global Infectious Disease Analysis, Department of Infectious Disease Epidemiology, School of Public Health, Imperial College London, London, United Kingdom

**Keywords:** Marburg, Marburgvirus, Filovirus, Vaccination, Vaccine trial

## Abstract

**Background:**

Outbreaks of Marburg virus disease (MVD) are rare and small in size, with only 18 recorded outbreaks since 1967, only two of which involved more than 100 cases. It has been proposed, therefore, that Phase 3 trials for MVD vaccines should be held open over multiple outbreaks until sufficient end points accrue to enable vaccine efficacy (VE) to be calculated. Here we estimate how many outbreaks might be needed for VE to be estimated.

**Methods:**

We adapt a mathematical model of MVD transmission to simulate a Phase 3 individually randomised placebo controlled vaccine trial. We assume in the base case that vaccine efficacy is 70% and that 50% of individuals in affected areas are enrolled into the trial (1:1 randomisation). We further assume that the vaccine trial starts two weeks after public health interventions are put in place and that cases occurring within 10 days of vaccination are not included in VE calculations.

**Results:**

The median size of simulated outbreaks was 2 cases. Only 0.3% of simulated outbreaks were predicted to have more than 100 MVD cases. 95% of simulated outbreaks terminated before cases accrued in the placebo and vaccine arms. Therefore the number of outbreaks required to estimate VE was large: after 100 outbreaks, the estimated VE was 69% but with considerable uncertainty (95% CIs: 0%−100%) while the estimated VE after 200 outbreaks was 67% (95% CIs: 42%−85%). Altering base-case assumptions made little difference to the findings. In a sensitivity analysis, increasing $R_{0}$ by 25% and 50% led to an estimated VE after 200 outbreaks of 69% (95% CIs: 53–85%) and 70% (95% CIs: 59–82%), respectively.

**Conclusions:**

It is unlikely that the efficacy of any candidate vaccine can be calculated before more MVD outbreaks have occurred than have been recorded to date. This is because MVD outbreaks tend to be small, public health interventions have been historically effective at reducing transmission, and vaccine trials are only likely to start after these interventions are already in place. Hence, it is expected that outbreaks will terminate before, or shortly after, cases start to accrue in the vaccine and placebo arms.

## Introduction

Marburg virus disease (MVD) is an acute, highly pathogenic, zoonotic, haemorrhagic disease caused by infection with Marburg virus. The wild reservoir of the Marburg virus is the Egyptian fruit bat (*Rousettus aegyptiacus*), which has a wide geographical range covering many parts of sub-Saharan Africa and the Middle East [1]. The virus was first discovered in a laboratory-derived outbreak in Marburg, West Germany in 1967 [2]. Since then, there have been 17 other spillover human infections or outbreaks of MVD, most of which have occurred in Sub-Saharan Africa, often associated with exposure to bats in caves or mines [3]. Human-to-human transmission is possible and whilst most outbreaks have been small, two outbreaks (one in DRC in 1998–2000 and one in Angola in 2004–2005) resulted in hundreds of cases and many deaths [4], [5].

The high pathogenicity associated with MVD and its potential to spread and cause public health emergencies has led the World Health Organization (WHO) to designate MVD as a priority for research and development into new vaccines, therapeutics and diagnostics [6]. Accordingly, there is now a number of MVD vaccine candidates which are in pre-clinical testing and one (a Chimpanzee Adenovirus vectored vaccine produced by the Sabin Vaccine Institute) which has completed a Phase 1 trial in humans [7] with Phase 2 trials planned for 2023. It is widely acknowledged, however, that Phase 3 efficacy trials for MVD vaccines will be very challenging to conduct due to the sporadic nature of MVD outbreaks which may occur over a large geographical range, the relatively small size of most outbreaks, and the necessity to control them as rapidly as possible using existing public health and infection control measures. Any Phase 3 vaccine trial will therefore have to deploy quickly to an affected area and even then the epidemic may end before sufficient cases have accumulated to determine vaccine efficacy with a degree of statistical confidence. Given the scarcity of MVD outbreaks there may be pressure to evaluate a number of available vaccine candidates simultaneously, with the potential of further eroding statistical power if different trials competed for eligible participants during an outbreak. To help negate these problems, an endpoint-driven platform trial design has been proposed [8] under a master protocol approach which could potentially remain open for recruitment over multiple outbreaks until sufficient endpoints have accrued.

Nevertheless, questions remain: how many outbreaks might be needed to evaluate an MVD vaccine, and thus, given the low frequency of MVD outbreaks, how feasible are Phase 3 trials likely to be? This study aims to answer these questions by utilising a mathematical model of MVD, which was parameterised based on a systematic review of data from all Marburg outbreaks up to and including the outbreak in Guinea in 2021 (comprising 15 of the 18 known MVD outbreaks) [3]. The model is used to simulate an hypothetical individually randomised Phase 3 MVD vaccine trial to determine how many cases and outbreaks might be required to determine vaccine efficacy (VE).

## Methods

We used a branching process model previously developed to simulate MVD transmission over time. New infections generated at any time *t* are governed by the force of infection *λ_t_*, which is determined by previous case incidence *y_s_* (*s* = 1, …, *t*-1), the serial interval distribution (denoted by *w*, its probability mass function), and the reproduction numbers *R_s_* as:

(1) $$\lambda_{t}=\sum s=1,...,t-1\;R_{s}Y_{s}W(t-s)$$

New secondary cases at time *t* are then drawn from a Poisson distribution so that:

(2) *y_t_* ∼ Poisson(λ*_t_*)

Eq. (1) shows that the reproduction number *R_s_* is allowed to vary over time. This is used to distinguish, in any given outbreak, three phases: a first one, during which transmission is maximum (*R_s_* = *R*_0_, the basic reproduction number), a second one during which non-pharmaceutical intervention reduces transmission by a factor *E*, the intervention efficacy, so that:

(3) *R_s_* = *R*_0_ (1 - *E*)

and finally, a third during which vaccination further reduces transmission by a factor *V*, the vaccine efficacy, so that:

(4) *R_s_* = *R*_0_ (1 - *E*) (1 - *V*)

Intervention is defined, in this context, as the implementation of measures such as case isolation, contact tracing and barrier nursing. Vaccination is assumed to occur after intervention and so we modified the model to include a delay from intervention to vaccination.

**Fig. A1:**
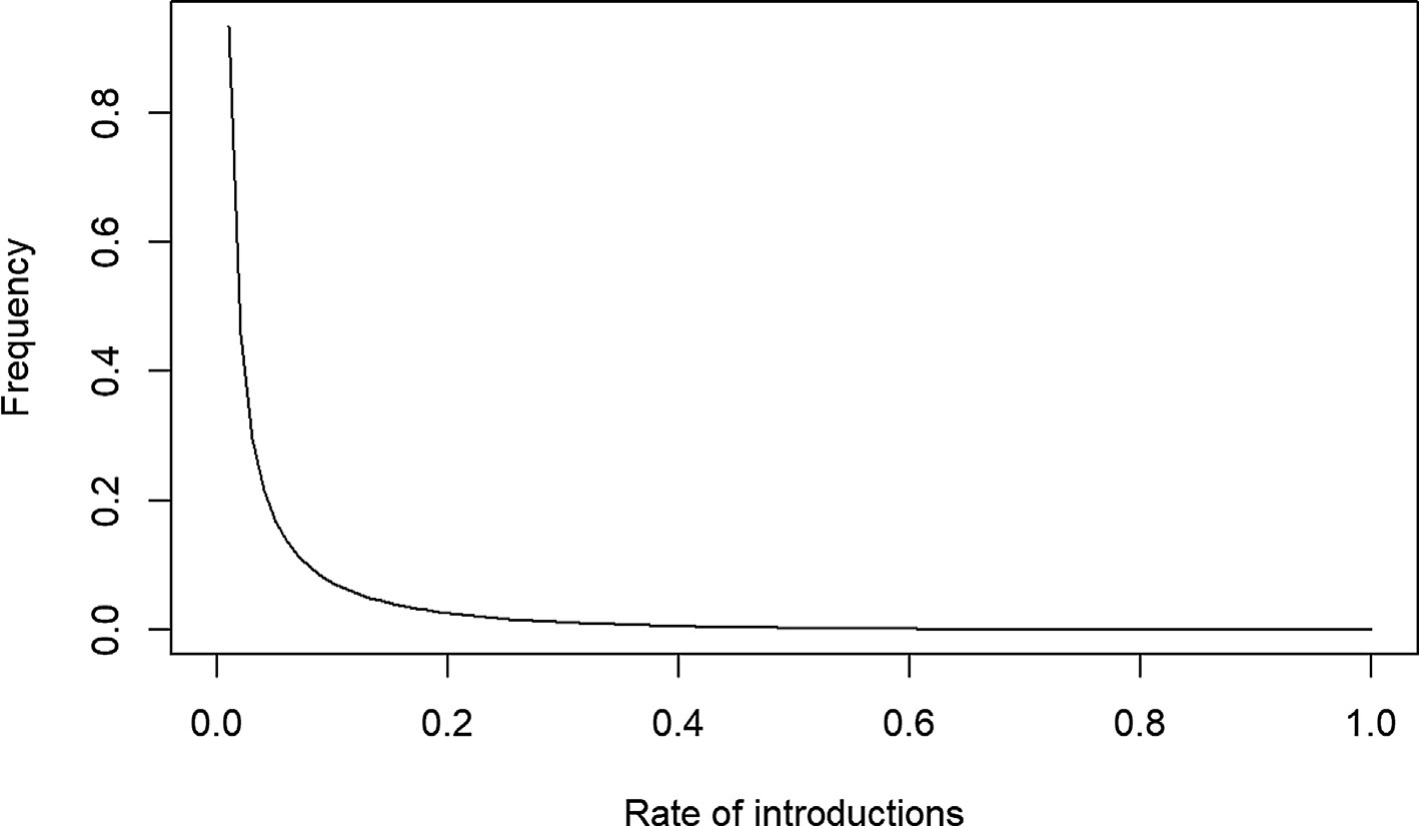
Plot showing the beta distribution of the rate of introductions per day used in our simulations.

Using this model, we simulated a trial involving a candidate vaccine with a nominal vaccine efficacy (VE) of 70% in the base case. We assumed a reactive mass vaccination strategy with 50% trial coverage in affected areas in the base case [9]. Across the 15 previous MVD outbreaks that we examined, the median delay between onset of the first case and beginning of interventions was 21 days [3]. We assumed that 2 further weeks were required for a vaccination campaign to be implemented in the base case. Hence, interventions and mass vaccination were simulated 21 and 35 days, respectively, after the first case. The time for vaccine efficacy to peak was assumed to be 7 days [see [3]]. We assumed the rate of zoonotic introductions followed a beta distribution with parameters α=0.01 and β=4. Such a distribution led to around 95% of simulated outbreaks having 1 introduction per year, which reflects the fact that most recorded outbreaks have had only one introduction and ended within a year of detection (see Appendix, Figure A1).

Trial participants were divided into 2 groups: one whose participants received the vaccine (25% of the population) and another who received a placebo (25% of the population). The other 50% of the population were assumed not to be enrolled in the trial, for various reasons *e.g.* ineligibility, refusal to consent, being absent on the day of enrolment etc. [9]. We varied the fraction enrolled in the trial in the sensitivity analysis by simulating both 30% and 70% coverage. We used our branching process model with mass vaccination, described above and in [3] to simulate 5000 outbreaks of MVD. For each outbreak, we reported the total number of cases as well as the number of cases in the placebo and vaccine arms. Subsequently, we calculated the vaccination efficacies [10] and associated 95% confidence intervals for sets of 10, 20, 30 etc. outbreaks, using 500 bootstrap samples to account for the variation in outbreak sizes across simulations. Vaccine efficacy was defined as:

(5) $$\mathrm{VE}=1-\frac{\mathrm{Number}\;of\;cases\;in\;vaccine\;arm}{\mathrm{Total}\;number\;in\;vaccine\;arm}/\frac{\mathrm{Number}\;\;of\;cases\;in\;placebo\;arm}{\mathrm{Total}\;number\;in\;placebo\;arm}=1-\frac{\mathrm{Number}\;\;of\;cases\;that\;are\;vaccinated}{\mathrm{Number}\;of\;cases\;in\;placebo\;arm}$$

The second equality is due to having a balanced population in the placebo and vaccine arms. We also estimated the number of outbreaks required to reach 150 cases. This was previously calculated as the required number of cases to provide 90% power to reject the hypothesis that VE would be less than 30% when its true VE was at least 60% [8]. It is worth noting that any of these values – the 30% null in particular – can be altered to give a lower number of cases required. Moreover, interim analysis should occur at 50 and 100 cases and so can be used to reject earlier*.* However, in this paper we will keep to 150 required cases.

Cases were included in the calculation of vaccine efficacy only if they occurred at least 10 days after vaccination, as it is assumed that there would be a delay between vaccination and the onset of immunity [8]. Due to relatively low transmissibility of MVD and high efficacy of non-pharmaceutical interventions (NPIs) [3], some simulated outbreaks may be controlled before having any case in both the vaccine and placebo arms. These outbreaks were ignored for VE calculations, but our results reporting VE estimations by numbers of outbreaks were corrected for the proportion of outbreaks controlled solely with NPIs.

To account for the uncertainty in some key parameters, we also carried out a sensitivity analysis, by changing the delay from the first MVD case to vaccination (from 35 days to 14 and 90 days), the vaccine coverage (from 50% to 30% and 70%), nominal efficacy (from 50% to 30% and 70%) and time from vaccination to infection in those that are infected (from a median of 9 days to 20 days). We also simulated a ‘best-case’ scenario in which we decreased the delay from the first MVD case to vaccination to 14 days, increased the coverage to 70%, and increased the time from vaccination to infection to a median of 20 days. Finally, we consider the effect of increasing the reproduction number, $R_{0}$, by 25% and 50%, as well as a combination of the ‘best-case’ scenario combined with a 50% increase in $R_{0}$.

## Results

Given a vaccine with a nominal 70% VE, and a vaccination campaign beginning 35 days after the first case was detected, we found that the median outbreak size was 2 cases, and the maximum 323. Only 0.3% of simulated outbreaks were predicted to have more than 100 MVD cases. For the vast majority (97%) of simulated outbreaks, there were no cases in the vaccine arm, *i.e.* the majority of outbreaks would be controlled in the absence of a vaccine.

Furthermore, in 95% of all outbreaks, there were no cases in both the placebo and vaccine arms. The largest outbreak (323 cases) had 10 and 36 cases in its vaccine and placebo arms, respectively.

**Fig. 1:**
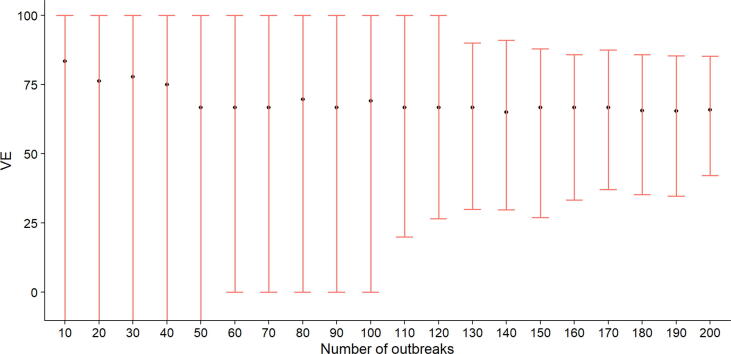
Vaccine efficacies and associated 95% bootstrapped confidence intervals calculated after different numbers of outbreaks (including those where there were 0 cases in both the vaccine and placebo arms). The lower CIs of the first 50 outbreaks were estimated to be negative.

Fig. 1 shows the estimated vaccine efficacies and their 95% CIs as a function of the number of outbreaks, including those where there were zero cases in both the vaccine and placebo arms. When VE was calculated using fewer than 50 outbreaks, the median VE ranged from 68 to 73%, although the associated confidence intervals were wide (95% CIs: -∞%- 100%). Note that VE can be -∞ if there are no cases in the placebo group but at least one case in the vaccine group, given equal numbers in the two arms. After 100 outbreaks, the VE estimate was 69% (95% CIs: 0%−100%) while the estimated efficacy after 200 outbreaks was 67% (95% CIs: 42%−85%).

Table 1 shows the variation in estimated VE from our sensitivity analyses (adjusting the delay to vaccination, coverage and nominal vaccine efficacy). Across all scenarios where we changed only one parameter, confidence intervals for VE were -∞-100% after 10 outbreaks and remained wide even after 100 outbreaks (typically 0–100%). In general, scenarios involving a higher coverage, lower VE, earlier intervention/vaccination and a lengthier time between vaccination and infection reduced the CIs slightly. These scenarios also took fewer outbreaks to reach 150 cases in the vaccinated and placebo arms (see Table 2). In particular, lengthening the delay between vaccination and infection reduced the number of outbreaks required to reach 150 cases to 387 (95% CIs: 156–696). Moreover, a combination of these scenarios (the ‘best-case’ scenario) reduced the CIs of the VE estimates (57–80% after 200 outbreaks), as well as the number of outbreaks required to reach 150 cases (264 outbreaks (95% CIs: 107–490)) even further. Finally, increasing $R_{0}$ by 25% and 50%, reduces both the CIs of the VE estimates (69% (95% CIs: 53–85%) and 70% (95% CIs: 59–82%) after 200 outbreaks, respectively) as well as the number of outbreaks required to reach 150 cases (452 (95% CIs: 195–781) and 315 (95% CIs: 172–482) after a 25% and 50% increase, respectively).

**Table 1:** Estimated median vaccine efficacy (VE) with 95% CIs (in brackets) calculated after 10, 20, 50, 100 and 200 simulated outbreaks, by varying the vaccination coverage, nominal VE and intervention/vaccination times. Baseline values: 50% coverage, 70% nominal VE, intervention and vaccination at 21 and 35 days, respectively.

Number of outbreaks	10	20	50	100	200
Baseline	83% (-∞-100%)	76% (-∞-100%)	67% (-∞-100%)	69% (0–100%)	67% (42–85%)
Higher Coverage (70%)	82% (-∞-100%)	75% (-∞-100%)	70% (-50–100%)	67% (0–100%)	67% (47–89%)
Lower Coverage (30%)	100% (-∞-100%)	87% (-∞-100%)	78% (-∞-100%)	76% (0–100%)	72% (31–100%)
Higher Vaccine Efficacy (90%)	100% (-∞-100%)	100% (0–100%)	100% (0–100%)	88% (50–100%)	89% (70–100%)
Lower Vaccine Efficacy (50%)	55% (-∞-100%)	52% (-∞-100%)	53% (-200–100%)	52% (0–83%)	52% (21–73%)
Later intervention and vaccination times (90 days after first case)	73% (-∞-100%)	75% (-∞-100%)	67% (-270–100%)	67% (0–91%)	67% (33–86%)
Earlier intervention and vaccination times (14 days after first case)	74% (-∞-100%)	70% (-∞-100%)	69% (-50%-100%)	67% (0–87%)	67% (43–80%)
Longer delay from vaccination to infection (mean: 20 days)	78% (-∞-100%)	74% (-∞-100%)	72% (0–100%)	71% (50–93%)	71% (58–84%)
R increases by 25%	79% (-∞-100%)	75% (-∞- 100%)	71% (0–100%)	72% (39–92%)	69% (53–85%)
R increases by 50%	75% (-∞-100%)	75% (-100–100%)	72% (29–93%)	71% (47–86%)	70% (59–82%)
Optimistic scenario, same R	79% (-∞-100%)	71% (-4–100%)	69% (33–100%)	71% (48–86%)	70% (57–80%)
Optimistic scenario, R increases by 50%	75% (0–100%)	71% (0–100%)	72% (50–87%)	71% (58–82%)	70% (63–78%)

* The optimistic scenario involves a combination of: longer delay from vaccination to infection, higher coverage, earlier intervention and vaccination but retaining a 70% nominal VE.

**Table 2:** Median number of MVD cases overall, median number of cases in both arms and average number of outbreaks required to exceed 150 cases in both arms, with 95% CIs (in brackets) under different scenarios.

	All cases	Cases in vaccine and placebo arms	Outbreaks required to exceed 150 cases
Baseline	2 (1–27)	0 (0–2)	767 (373–1225)
Higher Coverage (70%)	2 (1–30)	0 (0–2)	758 (385–1243)
Lower Coverage (30%)	2 (1–33)	0 (0–1)	887 (482–1392)
Higher Vaccine Efficacy (90%)	2 (1–27)	0 (0–1)	1011 (532–1604)
Lower Vaccine Efficacy (50%)	2 (1–29)	0 (0–2)	622 (337–983)
Later intervention and vaccination times (90 days after first case)	2 (1–79)	0 (0–2)	797 (448–1190)
Earlier intervention and vaccination times (14 days after first case)	2 (1–16)	0 (0–2)	687 (391–1059)
Longer delay from vaccination to infection (mean: 20 days)	2 (1–23)	0 (0–2)	387 (156–696)
R increases by 25%	3 (1–43)	0 (0–4)	452 (195–781)
R increases by 50%	4 (1–70)	0 (0–5)	315 (172–482)
Optimistic scenario*	2 (1–15)	0 (0–4)	264 (107–490)
Optimistic scenario, R increases by 50%	4 (1–30)	0 (0–9)	137 (27–251)

*The optimistic scenario involves a combination of: longer delay from vaccination to infection, higher coverage, earlier intervention and vaccination but retaining a 70% nominal VE.

Simulating a combination of the best-case scenario together with a 50% increase in $R_{0}$ reduced the CIs of the VE estimates even further (70% (95% CIs: 63–78%)) and required 137 (27–251) outbreaks to reach 150 cases.

## Discussion

It is unlikely that the efficacy of any candidate vaccine could be calculated before more MVD outbreaks have occurred than have been recorded to date. Our results suggest that the vast majority of future outbreaks may have no cases in both the vaccine and placebo arms, and would in fact be controlled before VE estimations could begin (ten days after implementing the vaccination campaign, which is when cases are included in any VE calculations). NPIs were sufficient to control 95% of simulated outbreaks. This is consistent with historical data: although MVD has a high case-fatality ratio, it is a rare and sporadic disease, with only 18 known outbreaks since 1967, and a low but variable reproduction number (varying across outbreaks from 0.5 [95% CI: 0.05 – 1.8] to 1.2 [95% CI: 1.0 – 1.9]), becoming substantially lower once NPIs have started (varying across outbreaks from 0.2 [95% CI: 0.006 – 0.7] to 0.6 [95% CI: 0.03–1.5]) (3). Additionally, any candidate vaccine itself would offer protection against MVD symptoms (70% VE in the base case), further limiting the size of any outbreak.

**Fig. 2:**
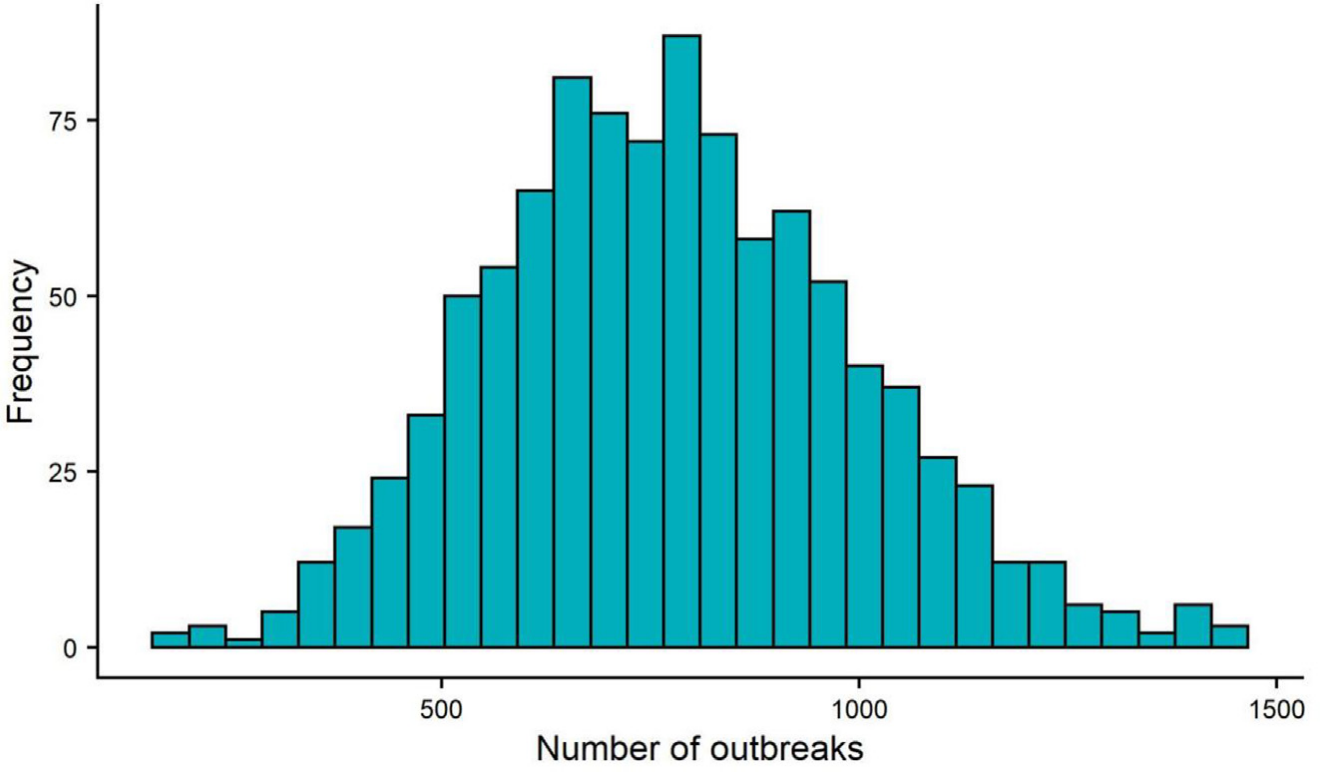
Number of simulated outbreaks required to reach 150 total cases for the vaccine and placebo arms, based on 1000 simulations.

Fig. 2 shows a histogram of the number of simulated MVD outbreaks required to reach 150 combined cases in the vaccine and placebo arms. As mentioned before, this is not a number that is set in stone, particularly as interim analyses can be used to reject earlier, but here we keep to the underlying assumptions of 90% power with a 30% null and no rejection occurring in interim analyses. The median number of outbreaks under baseline conditions was 767 and even in the best-case scenario (assuming no change in $R_{0}$), 264 outbreaks were required, which is an order of magnitude higher than the 18 MVD outbreaks recorded to date.

Furthermore, while there were over 150 cases in two previous outbreaks (154 in DRC, 1999–2000 and 374 in Angola, 2004-5), a large proportion of these cases occurred before effective interventions had taken place (82 and 142 cases, respectively) [3]. In a clinical trial, cases occurring before interventions would not be included in VE calculations, since the vaccination trial would not yet have been set up. Table A1 (Appendix) shows the number of cases from the 18 recorded MVD outbreaks, both overall and after interventions were put in place. 12 outbreaks had either 0 or 1 case after intervention, while 2 further outbreaks had 5 cases post-interventions. Only the 2 large outbreaks (DRC and Angola) had more than 5 cases post intervention, and these were likely due to repeated zoonotic transmission in DRC [4] or delayed adherence to the intervention programme in Angola [5]. Finally, detection of MVD outbreaks and intervention efficacy have improved since these two outbreaks [11], suggesting that future outbreaks may be more quickly contained than in the past.

Our sensitivity analyses (see Table 1, Table 2) show that while varying parameters such as an increased coverage rate, decreased time to intervention/vaccination and, in particular, lengthening the delay from vaccination to infection did reduce the number of outbreaks required to accurately determine VE, this number was still in the hundreds, on average. Even after 200 outbreaks, the difference between the upper and lower confidence intervals ranged from 15 to 70%, depending on the scenario simulated. Moreover, although simulating an increase in the reproduction number, $R_{0},$ also led to a lower number of outbreaks required, particularly when combined with other factors (increased vaccination coverage etc.), even a 50% increase in $R_{0}$ still required many more MVD outbreaks than have currently been detected. These CIs reflect the very large heterogeneity in outbreak size distribution and reinforce our message that obtaining the VE of any candidate MVD vaccine will be difficult in a Phase 3 trial, even if using the master protocol approach [8].

**Fig. A2:**
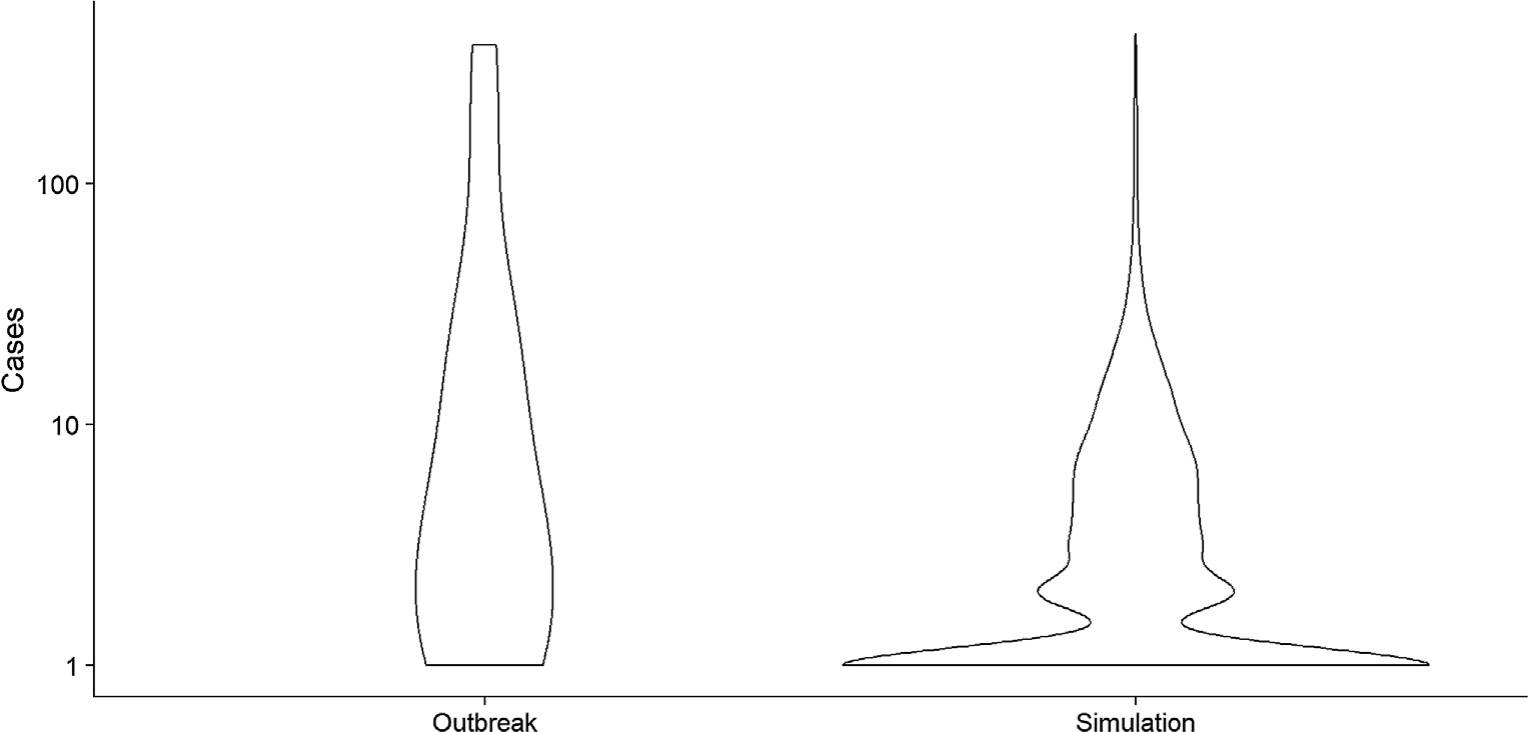
Violin plot comparing the number of cases observed during MVD outbreaks to date with cases estimated through model simulations.

**Fig. A3:**
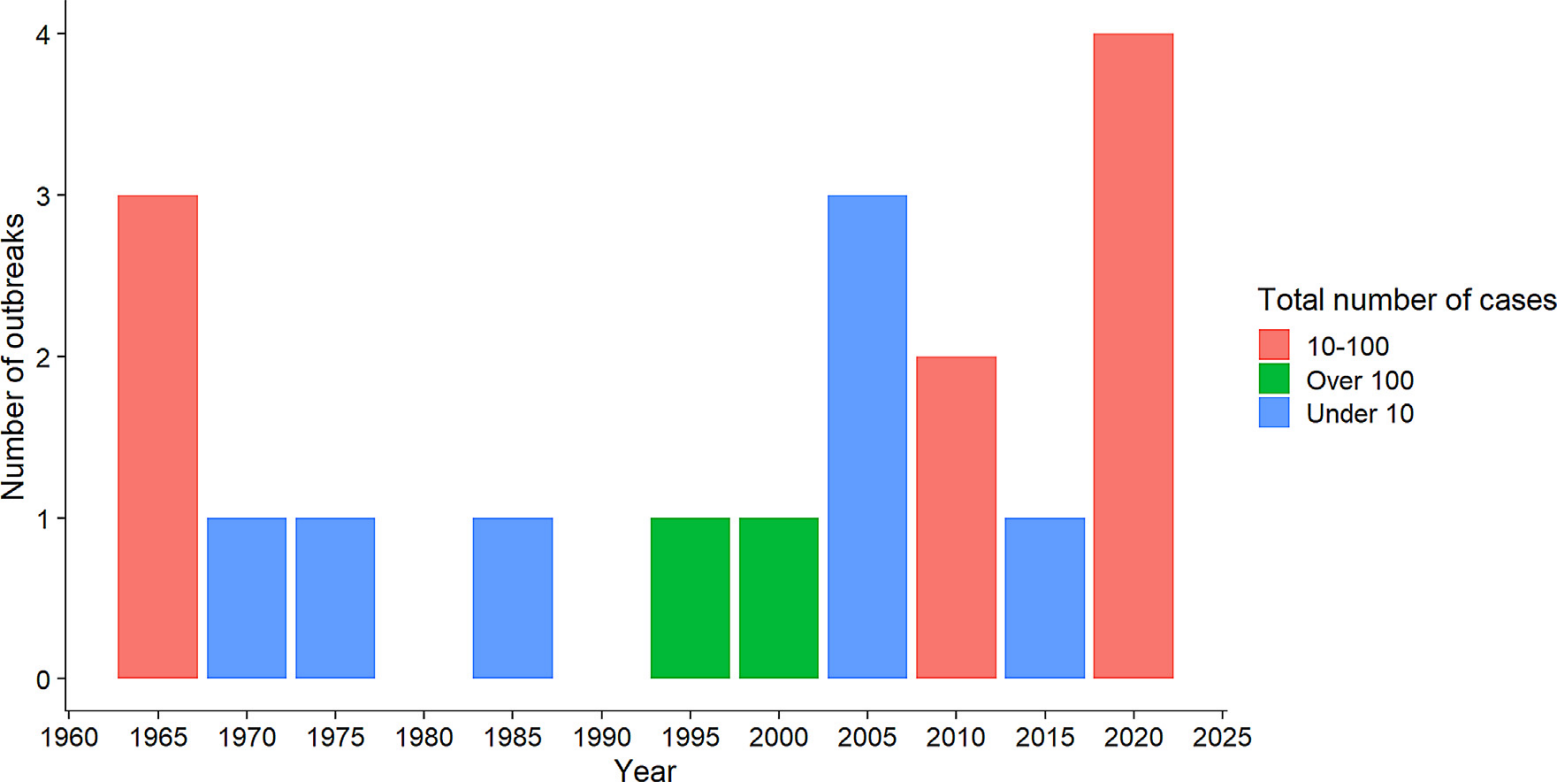
Bar plot showing the number of MVD outbreaks every five years since the first known outbreak in 1967, as well as the total number of cases from outbreaks during those years.

**Fig. A4:**
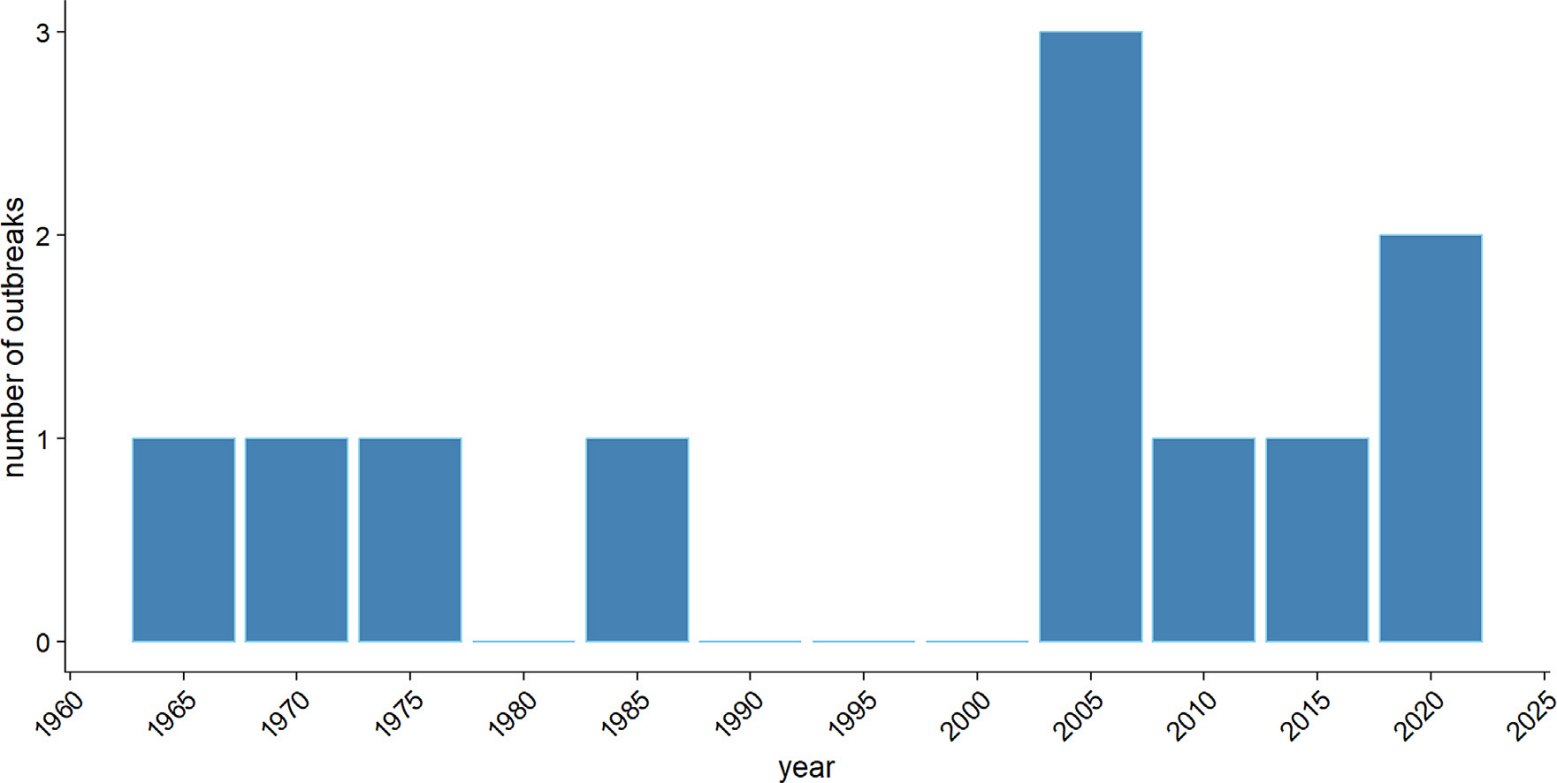
Bar plot showing the number of MVD outbreaks with fewer than 5 cases during every five years since the first known outbreak in 1967.

It is informative to map the projected number of outbreaks required to estimate VE onto time. This is not straightforward, however, as we would need to consider past outbreaks that have been missed, as well as whether surveillance has improved over time. At the time of writing, 18 outbreaks have been detected, with 2 currently ongoing (Table A1). Based on the latest reports, there appear to have been over 5 cases in both of these ongoing outbreaks. Hence, we will use information on case numbers for all 18 outbreaks in this particular analysis. Figure A2 compares the number of cases observed during previous MVD outbreaks with cases from model simulations. There is evidence from this figure that smaller outbreaks (fewer than 5 cases) have occurred in the past but remained undetected. Figure A3 shows the number of outbreaks every five years since 1967, as well as the total number of cases from outbreaks during those years. Figure A4, moreover, shows the number of outbreaks every five years with fewer than 5 cases. These two figures suggest that surveillance of MVD outbreaks – even those with fewer cases - has likely improved over time. Hence, to map the number of outbreaks onto time, we assumed in the first instance that the rate of outbreaks detected recently – 4 outbreaks over the last 5 years (Table A1) – would continue in future. Projecting this rate forward, we estimate that the 767 outbreaks required under baseline conditions (on average) would be detected after 960 years. The 137 outbreaks under the best-case scenario the best-case scenario, including a 50% increase in $R_{0}$, would require 170 years to be detected. As a sensitivity analysis, if the rate of detection were to double to 8 outbreaks per 5 years, reflecting improved surveillance, detection would take 480 and 85 years for the baseline and best-case scenarios, respectively.

There are several limitations to our study. First, no licensed vaccine yet exists and so parameters such as the nominal VE are indicative only. There is also a general paucity of data available on the epidemiology of MVD and the effectiveness of public health interventions designed to reduce its spread, due to the sporadic nature of MVD outbreaks. In addition, we assume that both interventions and vaccine trials were implemented across all affected areas immediately, whereas in reality they may be rolled out over time. Using time-varying functions might help improve the accuracy somewhat, but there is a lack of data to inform the modelling of these functions. While this would decrease the number of outbreaks required for estimating VE, our conclusion that a prohibitively large number of outbreaks would be required is almost certainly robust to such changes. Moreover, it is worth noting that, if a clinical trial were to be set up, a ring vaccination approach with intermediate and delayed rings, without a placebo, would likely be used instead of the individually randomised approach that we have modelled here. Cluster randomised trials are less efficient than individual-randomised trials due to intra-cluster correlation. Therefore, such trials would require more cases and, therefore, outbreaks. The ring vaccination approach can target the trial to areas of active transmission when an outbreak is ongoing, reducing the number of required vaccine doses but does so at the cost of reduced power. Finally, it is plausible that human incursions into bat habitats may become more frequent, leading to more frequent outbreaks of MVD. This would decrease the time needed to achieve VE in a multi-outbreak trial, but not the number of outbreaks required.

## Conclusions

Our simulations suggest that a Phase 3 vaccine trial run under the master protocol approach where endpoints are accumulated over multiple outbreaks, would likely require a large number of outbreaks to accurately estimate vaccine efficacy - more outbreaks than have been observed since MVD was first discovered in 1967.

## Declaration of Competing Interest

The authors declare the following financial interests/personal relationships which may be considered as potential competing interests: W John Edmunds reports financial support was provided by Department of Health and Social Care. Thibaut Jombart reports financial support was provided by MRC Centre for Global Infectious Disease Analysis. W John Edmunds reports financial support was provided by Japan Agency for Medical Research and Development. George Qian reports a relationship with Pfizer that includes: funding grants and non-financial support. GQ works on a separate project that is funded by Pfizer (please see ’Other Support’ section).

## Figures and Tables

**Table A1:** Marburgvirus outbreaks since 1967, including the number of cases both overall and after interventions were put in place.

Outbreak Location	Year(s)	Total number of cases	Cases after intervention
Marburg, Germany	1967	24	5
Frankfurt, Germany	1967	6	1
Belgrade, Yugoslavia	1967	2	1
Johannesburg, South Africa	1975	3	1
Nairobi, Kenya	1980	2	0
Nairobi, Kenya	1987	1	0
Durba and Watsa, Democratic Republic of Congo	1998–2000	154	72
Uige, Angola	2004–2005	374	232*
Uganda	2007	4	1
USA (via Uganda)	2008	1	0
Netherlands (via Uganda)	2008	1	0
Uganda	2012	26	5
Uganda	2014	1	0
Uganda	2017	4	0
Guinea	2021	1	0
Ghana	2022	<5	1
Equatorial Guinea**	2023	8	TBC
Tanzania**	2023	23	TBC

* The interventions in Angola were reportedly slow to be adopted. ** As of May 2023, the outbreaks in Equatorial Guinea and Tanzania are ongoing. Case numbers are based on reports from March 2023.

## Data Availability

Data will be made available on request.

## Appendix

See Fig. A1, Fig. A2, Fig. A3, Fig. A4 and Table A1.
